# Culling and mortality of dairy cows: why it happens and how it can be mitigated

**DOI:** 10.12688/f1000research.55519.1

**Published:** 2021-10-06

**Authors:** Diniso Simamkele Yanga, Ishmael Festus Jaja

**Affiliations:** 1 Department of Livestock and Pasture Sciences, University of Fort Hare, Alice, Eastern Cape, 5700, South Africa

**Keywords:** Milk production, culling, mortality, dairy animals, lameness, mastitis

## Abstract

The United Nations estimates that the global population will total 9.7 billion in 2050. Rapid population growth pose a significant obstacle to achieving the Sustainable Development Goals, particularly eradicating hunger and poverty. In view of the expanding population growth, food production ideally should triple to prevent massive food shortages. Sustainable food and nutrition security is the focal point of the dairy industry. Dairy production plays a pivotal role in addressing and advancing global food and nutrition security. It serves as a major source of protein, calcium, and phosphorus in many families in developing countries with a fast-growing population. Consequently, the dairy industry is expected to grow by approximately 26% in the next 10 years and produce an estimated 1077 million tonnes of milk by 2050. However, the growth and distribution of the dairy industry is limited by many factors such as culling and mortality of dairy cows. Several studies highlight reproduction failures, old age, poor milk yield, diseases (mastitis, lameness, and dystocia), and heat stress as some reasons for culling of dairy cows. Hence, this review highlights the factors influencing culling and mortality in dairy production farms, and discusses mitigating measures to limit culling.

## Introduction

Globally, the growth and distribution of the dairy industry is anticipated to rise by 26% within the next 10 years. ^1^ In some countries like South Africa, there has been a steady growth of 4.8% in 2018 and 3.0% in 2017. ^2^ There is an expected production of 1077 million tonnes of milk by 2050. ^3^ The projected rise will be escalated by, but not limited to, the rapid growth of the maize production industry, which supplies the dairy industry with feed. The availability and access to nutritious dairy products will also increase with the industry's growth and distribution. Increased availability and access will aid in enhancing food security, especially in developing continents. However, the demand for milk and milk-related products far exceeds the current supply of milk and milk-related products and also exceeds the expected 26% rise. ^1^^,^^4^^,^^5^ For instance, South Africa experiences a 3,500,000 litre milk deficit every year. ^6^ In 2016, South Africa contributed 0.5% to the overall global milk production. ^7^ The general imbalance in the demand and supply of milk is mainly due to the escalating global population growth. ^4^

Among many factors influencing milk quantities and qualities, culling and mortality of cows have been highlighted as significant dairy industry constraints. Culling is mainly divided into voluntary and involuntary culling. Involuntary culling accounts for most reasons why dairy animals are removed from production. ^8^^–^^10^ Culling is also regarded as a damage control and preventative strategy in which the farmer eliminates cows that carry undesired traits and defects. ^10^ Culling also aids incorporating replacement heifers (the availability of a replacement heifer with superior potential influences the decision to cull a cow regardless of other factors ^10^) and maintaining an optimum herd size. Dairy cows can be removed during production, such as the lactation stage, and culling records are essential in drafting a herd health program. ^9^^,^^11^ Culling, to some extent, mitigates mortality as cows that are injured or prone to diseases are most likely to be eliminated through market channels, such as sales and auctions. Documenting the factors responsible for culling helps to identify several problems affecting the farm from cow-level to herd-level and from a managerial to economic point of view. ^8^^,^^10^^,^^12^^–^^15^ Understanding culling rates and related factors is of utmost importance as it is often associated with managerial expertise. ^8^^,^^15^ At the herd level, culling is influenced by several factors such as replacement heifers plan, milk quotas, and market prices of milk and beef. ^15^^,^^16^ The most common reasons for culling at cow-level includes old age, diseases (udder and legs), metabolic diseases or disorders, respiratory diseases, infectious and non-infectious diseases, illness, injury, infertility, and accidents. ^17^^,^^18^ There are several studies conducted in several countries in the world on culling effects and culling factors except in Africa. ^19^^–^^22^ There is limited, or lack thereof, information on the culling strategies and rates in Africa. Hence, this review seeks to highlight the factors responsible for culling and mortality in dairy farms.

## Literature search

Studies covering any of the factors responsible for culling and mortality in dairy production farms were targeted and included in this review. These studies did need not be addressing the factor in relation to culling and mortality per se to be included in the review. Peer-reviewed literature were identified using databases such as WHO Library Information Systems (WHOLIS), Web of Science, Science Direct, Google Scholar, Scopus, and Norwegian Register for Scientific Journals, Series and Publishers (NSD). Other databases searched were MEDLINE (NLM), Agriculture, Biology and Environmental Sciences (Clarivate Analytics), and PubMed Central (NLM). In addition, grey literature was searched using Google search engine. The following terms were used: culling, culling and mortality, dairy production, dairy industry, diseases, dairy animals, diseases of dairy production, predisposing factors of diseases in dairy production, lameness and associated factors. Also, we searched lists and citations of related studies. Various articles were identified and thoroughly read to extract common factors responsible for culling and mortality in dairy animals. The causative factors were grouped into several classes: udder-related (mastitis) reproduction, production, growth and leg factors, sales, health, and miscellaneous factors. ^8^ The search focused on both commercial and communal dairy farms. A summary of the studies included in this article can be found in Table 1.

**Table 1.** Summary of studies conducted on culling in dairy farms.

Continent	Country	Period	Farm type	Culling factors	No of animals	Breeds	Feeding system	Milking sessions	Reference
Europe	Italy	2001-2007	Commercial dairy farms	Heat Stress	191 012	Not specified	Not specified	Not specified	19
South America	New York	2003-2008	Commercial farms	Gram-negative and Gram-positive Clinical Mastitis	7 herds	Holstein	TMR	3	20
Asia	New Zealand	2007	Commercial	Mastitis	30 herds (708 heifers)	Friesland, Jersey and Cross	Pasture - ryegrass & clover	Not Specified	21
Asia	New Zealand	1990-2013	Commercial	Reproduction disorder, Production performance, Mastitis	9 411 385	Not Specified	Pasture - ryegrass & clover	Not Specified	23
America	United States	1993-99	Commercial	Reproduction, production, and mastitis	6264-19 464 cows	Not Specified	Not Specified	Not Specified	13
Europe	Poland	2006-2007	Commercial	Reproduction disorders and limb diseases	258 cows	Holstein	TMR	2	24
Europe	France	1989-1994	Commercial	Reproduction disorders and poor milk yield	59 herds	Holstein	Not Specified	Not Specified	25
Europe	England	1995	Commercial	Reproduction, mastitis, and production	Not specified	Holstein	Not Specified	Not Specified	12
Europe	United Kingdom	2008-2010	Commercial	Reproduction problems, Mastitis, and management policy	6 486 823	Not Specified	Not Specified	Not Specified	9
Europe	Ireland	2003-2006	Commercial	Reproduction/infertility, surplus and low milk yield	Not specified	Not Specified	Not Specified	Not Specified	26
America	United States	2001-2006	Commercial	Reproductive factors	727 herds	Mixed	Not Specified	Not Specified	27
America	United States	2018	Commercial	Milk price, cancer eye and milk production	9400 cows	Not Specified	Not Specified	Not Specified	22
Europe	England	1990-92	Commercial	Poor fertility, management practices, mastitis	Not specified	Holstein, Jersey and cross	Not Specified	Not Specified	28
America	America	Nov-09	Commercial	Low productivity, breeding and abortion.	Not specified	Holstein, Jersey and cross	Not Specified	Not Specified	29
America	New York	1994-95	Commercial	Diseases	Not specified	Holstein	Not specified	Not Specified	11
Asia	New Zealand	2014-15	Commercial	Lameness	823 cows	Holstein	Pasture - ryegrass & clover	Not Specified	30
Europe	Sweden	2004	Commercial	Lameness	2368 cows	Holstein	TMR	Not Specified	31

## Major factors responsible for culling and mortality

### Diseases

Animal diseases are responsible for a significant loss of production in dairy production farms. Diseases are seldom planned for, some cannot be eradicated, some are zoonotic, and hence they present a fatal risk to the whole herd and public health. Furthermore, some animal diseases are too costly to treat, ^11^^,^^32^ hence are further regarded as indirect causes of poor milk yield because sick animals lose appetite and are mostly depressed, and as such cannot yield the expected quantity of milk. ^11^^,^^33^ Important considerations are made before a decision to cull is taken, such as the physiological status of the cow, genetic variability and adaptability, and milk production potential. ^34^ Diseases have a direct influence on the culling and mortality rates of dairy cows. ^11^ Some of these diseases include bovine neosporosis caused by *Neospora caninum,* which affects intensive dairy production. In addition, other common diseases or disorders that play an important role in culling decisions include milk fever, lameness, dystocia, udder deformities, ketosis, metritis, and mastitis.

*Bovine neosporosis*

Bovine neosporosis causes abortions, stillbirths, and drops in milk production amongst dairy cows worldwide. ^32^ As such, a cow that has been diagnosed with *Neospora caninum* is culled from the herd. A study conducted in Senegal reported that sero-positive cows, when not culled, require more inseminations during breeding season than sero-negative cows resulting in more costs incurred. ^32^ It is, therefore, to a certain degree, logical and cost-effective to cull cows that are sero-positive for neosporosis.

*Milk fever and ketosis*

During early lactation, first 30 days, there is a high culling rate due to milk fever. ^11^ It has also been reported that milk fever also contributes to lactation culling, especially late lactation after 240 days. ^11^ The other disease of interest that has been identified as a causative agent for culling and mortality of dairy cows is ketosis. Ketosis refers to an energy imbalance in the cow in terms of the demand and available energy for the cow. ^11^^,^^32^ This disease requires a high level of management as it has resulted in the culling of many cows during late lactation. ^11^ Cows with ketosis are culled because they tend to disrupt the breeding process and, in some cases, results in infertility. ^11^ However, the effect of ketosis on culling of dairy cows has not been concluded as different studies have contradicting reports in terms of the risk level. Contrary to earlier studies, it has been reported that ketosis can be easily managed and has no effect on milk yield after 30 days in milk thus invalidating ketosis as a major factor responsible for culling. ^35^

*Mastitis*

Mastitis is the most significant disease of economic importance in the dairy industry. In clinical mastitis cases, the udder is characterised by inflammation, pain (the cow refuses to be milked), discolouration of milk, watery and bloody milk, flakes or clots to purulent exudate, and reddish discolouration of the skin and swelling of the udder. ^11^^,^^36^^–^^38^ The disease has been responsible for the culling of many dairy cows during the first and second lactation with a risk ratio of 1.9. ^11^ Evidence from many recent studies shows that mastitis increases the risk of culling in dairy cows. ^11^^,^^39^^,^^40^ The prevalence of mastitis varies with the cow’s stage of lactation; heifers are more susceptible to clinical mastitis immediately after calving. ^21^^,^^41^ Furthermore, cows that exhibit recurrence of clinical mastitis are most likely to be culled than first-time casualties as they become a liability to the farm. ^42^

Mastitis in dairy cows, at any milk production stage, may be induced by one pathogen or a combination of many pathogens. ^20^ Mastitis cases are classified in terms of the origin of the pathogen, i.e. contagious pathogens and environmental pathogens. ^43^ Examples of contagious pathogens are *Staphylococcus aureus, Streptococcus agalactiae, Corynebacterium bovis and Mycoplasma species*. ^37, 38, 43, 44^ Contagious pathogens are mainly found in the cow’s intra-mammary glands. They are transmitted from one cow to another through milking machines, flies, and milking operators’ hands. ^43^ Environmental pathogens originate from the cow’s surroundings and are transmitted into the cow through the teat during contact with contaminated water, contaminated bedding, and contaminated soil. ^43^^,^^44^ Examples of environmental pathogens are *Escherichia coli, Streptococcus uberis, Streptococcus dysgalactiae* and *Klebsiella* species.

The severity of infection caused by these pathogens varies, and depends on the stage of lactation, lactation number, age of the cow, and the type of breed. ^20^ These mechanical dynamics present a risk of lesions or alteration of the teat end, inducing subclinical mastitis in the process. ^44^ Gram-negative pathogens are accountable for clinical mastitis in high-producing multiparous cows. ^45^

In terms of cost, mastitis is a disease of economic importance in dairy farms as it directly influences the milk quality and quantity ^42^^,^^45^ which, when compromised, have significant financial implications. ^41^^,^^42^ Mastitis-related economic losses in a dairy farm include costs of medication, veterinarian costs, labour costs, loss of quality (drop in butterfat content), discarded milk, culling and occurrence of other diseases. ^46^ These economic losses depend on the type of production system; an intensive system incurs more costs due to the high-input production methods whereas an extensive system incurs the least costs. The economic losses also vary on the type of mastitis: clinical mastitis (associated with acute cases) and subclinical cases (chronic cases). In recent studies, economic losses due to mastitis have ranged from 2369.72 Rand (120 Euros) to 2765 Rand/cow/year (140 Euros). ^46^^,^^47^ In a study conducted in the Netherlands, there was approximately 107 kg and 336 kg of milk per lactating cow lost due to subclinical mastitis and clinical mastitis, respectively. ^47^ It is evident that clinical mastitis results in severe milk production losses even though it is associated with acute cases. Milk production losses also include discarded milk due to contamination, whether thrown away or fed to the calves. ^48^ In the first 30 days of a lactation study, discarded milk accounted for 5.7% of direct costs incurred in a dairy farm per case of clinical mastitis, approximately R450.00 (US$25) per case. ^48^ In the same study, veterinary costs were responsible for only 1% of the total direct costs due to mastitis. A similar trend was reported in similar studies. ^46^^–^^48^

Mastitis further causes significant losses through reproductive disruptions which, when they occur, means that the farmer is most likely forced to cull the cow. ^11^^,^^20^^,^^49^ Replacement heifers’ susceptibility and high incident rate to clinical mastitis peri-partum presents the farm with substantial financial losses in terms of medical costs and drop in milk yield. ^21^

### Lameness

Lameness is a paramount indicator of animal welfare; this is because lameness is a condition that is commonly associated with pain. ^50^^–^^52^ In the United States, lameness is noted as a significant contributor to the culling of dairy cows. ^53^ For dairy farmers, lameness is a sensitive subject because it imposes serious financial losses, as such culling becomes an alternative. Lameness is also sensitive to dairy farmers as it may be associated with the farm’s poor detection of locomotion defects. ^50^ In the United Kingdom, one study reported the prevalence of lameness as 36.5% in 205 dairy farms. ^50^ The study further emphasised that cases of lameness vary with seasons and environment in which the cows are exposed. There are more cases of lameness in spring. ^54^ Lameness is closely associated with an intensive milk production system, in which the cows are subjected to standing for long periods on a concrete floor, and imbalanced diets that solely are formulated to enhance milk production yet predispose the cow to lameness. ^52^

A study conducted in New Zealand reported that lameness is closely correlated to reproductive failures. In cases where lameness affects reproductive performance, such cows have increased chances of being culled. ^30^ This further buttresses that reproductive failures are mainly responsible for the culling decision in many dairy farms. ^9^^,^^23^^,^^25^ Lameness is most common in multiparous cows, especially cows that are in third or higher parity. Therefore, lameness coincides with peak production of the dairy cows, which is tricky for a farmer as replacement heifers would not immediately meet production levels required. ^8^^,^^30^ Lameness can be in the form of claw ulcers which are closely associated with milk yield or production performance. ^31^ Lameness is considered as a disease of economic importance as, upon occurrence, it affects the whole lactation period of the cow. As a result, culling due to lameness is a rationally taken decision in most cases. Lameness is a disease of economic importance in dairy farms; it significantly reduces milk yield. ^50^^,^^59^ Milk yield is the currency of a dairy farm. Thus, any loss in milk yield has a monetary value attached to it. Consequently, a cow with a persisting lameness case is at high risk of being culled. Lameness has also been closely linked to poor fertility amongst dairy cows. ^53^ Poor fertility is a major factor responsible for culling. ^25^ One of the effects of lameness is delayed ovarian cycles, which disrupt the breeding cycle ^53^ and eventually induce poor reproductive performance. ^31^ Dairy cows with a reduced reproductive performance are immediately culled from the dairy farm. ^15^ There is a positive correlation between delayed ovarian cycle and body condition losses, ketosis, and puerperal disturbances. Also, veterinary costs associated with the treatment and management of lameness increase the cost of managing and running dairy farms. ^30^ Furthermore, sub-optimal treatment of lameness results in high lameness recurrence cases. ^30^

The various factors that induce lameness are environment, management, the cow (breed, stage of lactation, age), and nutrition. ^51^ Furthermore, environmental factors such as grazing pastures, type of housing, and concrete surfaces are other predisposing factors of lameness. ^50^ Specifically, concrete surface is closely associated with hoof lesions. ^50^^,^^55^ There are more lameness (claw disorders) incidences amongst dairy cows that are housed indoors (concrete floors) than those that are under pasture-based systems, whereby they are frequently susceptible to small rocks, wire and metal. ^52^ A damaged hoof and joint stiffness impair a dairy cow’s locomotion, ^31^ directly affecting the cow’s feeding patterns. It is precisely for this reason that farmers must pay close attention to claw health. Impaired locomotion is more evident amongst Holstein-Friesland because of their high milk yield pedigree. ^56^ Bony changes in the pedal bone accounted for the increased vulnerability to the lameness of old dairy cows. ^30^

The nutritional disorder closely associated with lameness is clinical and subclinical ruminal acidosis. ^51^ Discrepancies in herd management often lead to nutrition disorders such as systemic or metabolic acidosis. ^31^ A concentrate diet fed with limited functional fibres results in reduced chewing, so the cow produces a limited quantity of buffering saliva, resulting in a drop of the rumen pH. ^57^ Hence the blood becomes acidic from too much acid being absorbed from the rumen into the blood, leading to acidosis.

Acidic blood cannot carry as much oxygen and cow’s feet, being at the farthest points of the cow’s body, receive the least oxygen. ^57^^,^^58^ The lack of supply of blood causes the release of histamine, a vasodilator, and arterial constrictor. The sensitive tissues lining the outside wall of the hoof and coffin bone cause ulcers, pain, and haemorrhages associated with laminitis. ^57^^,^^58^

### Poor reproduction performance

Several studies have reported a very close association between reproductive failures and high culling rates. ^9^^,^^15^^,^^23^ Reproductive failures have been closely linked to the age at first calving. Early age at calving improves reproductive performance. ^17^ Conception status is considered the key influencer in culling a cow. ^11^ Reproduction failures, specifically missing conception or failure to deliver the calf, are a leading global factor that causes the culling of dairy animals. ^9^^,^^13^^,^^23^^,^^25^ Cows that generally fail to conceive from a single service tend to have lengthy calving intervals. ^29^ Such cows that are costly to the farm, as a result, have a high probability of being culled than the cows that readily conceive.

*Heat stress*

Heat stress negatively interferes with production and reproduction dynamics. Hence it is of economic importance in the dairy production industry. ^60^^–^^62^ The oestrus cycle and the conception rate of dairy cows are key factors that affect breeding outcomes in a particular breeding season. During periods of heat stress, cows are seldom likely to show signs of oestrus or heat, which is a further indication of decreased amounts of reproductive hormones in the blood. ^63^ Without signs of oestrus, the farmer does not know that a cow should be artificially inseminated or be bred, and when a cow does not get inseminated, the cow cannot conceive a pregnancy. Besides, heat stress disrupts the embryo's development by significantly reducing the cow’s dry matter intake, which is a critical component of gestating dairy cows. ^64^ It further distorts embryo development by inhibiting the functioning of dominant follicles and oocytes, thus rendering them unable to breed successfully.

*Infertility*

The goal of any breeding program should be to have 90-95% of cows bred in a 65-day breeding season. ^65^ Often, this is not the case due to nutritional and non-nutritional factors. Factors causing infertility and animals showing a diminished or absent capacity to produce viable offspring are removed from the breed stock. ^65^ In a study conducted in France, infertility (26.1%) was the most frequent cause for culling than low milk yield. ^25^ In the same study, more than 50% of other reasons were health-related such as lameness. ^50^ Poor fertility is a global dairy production challenge that has caused the culling of many dairy cows. For instance, in one study investigating culling in 50 dairy herds in England, poor fertility was responsible for 37% of culling. ^28^ A review conducted in Southern Africa amongst smallholder dairy farms mentioned that contributing factors to infertility includes inadequate housing facility, poor nutrition and improper health management systems. ^66^ Infertility results in poor herd growth, a decrease in replacement of heifers, and generally low farm productivity ^66^; hence it is logical to cull infertile cows as they become a liability to the farm. The unavailability of replacement heifers limits voluntary culling in which the farmer has the prerogative to cull dairy cows. ^17^^,^^18^ It further results in the retention of older cows even when the milk yield is declining.

*Parity*

Previous studies have shown a relationship between parity and the culling rate of cows. ^67^^,^^68^ An increase in parity is often associated with a continuous drop in culling frequency, with the highest culling rate at parity 1 to 3. ^67^ Animals in parity 1 to 3 produce more milk than animals in the other parities. Hence, culling dairy cows between first parity and third parity is a costly exercise for dairy farms because it is a stage whereby cows are producing more milk. ^18^^,^^67^ Udder morphological disorder is one of the leading predisposing factors for culling across parities. ^8^^,^^23^^,^^25^ However, culling due to udder problems is more evident during the fourth to sixth parity, where it accounts for almost 50% as a reason to cull. In contrast, reproductive failures are the main factors that lead to culling in second and third parities. ^29^ Parity presents a significant risk for culling, especially to Holstein-Friesian, more than other dairy breeds. ^29^ The first and second parity are two vulnerable stages in the cow’s production life as it is exposed to cases of mastitis and ketosis. ^11^ It is in these stages that culling due to ketosis is frequently reported.

### Culling due to age

Culling based on age varies with farm management styles/plans, herd size, and milk yield prowess. ^17^ There is an association between herd size and age at culling, in which the smallest herd size had the oldest age at culling. ^17^ Several studies have reported fewer incidences of culling due to age. Therefore, this low incidence rate of culling due to age is hypothetically an indicator of a lack of potential for longevity and lengthy productivity amongst dairy cows. The availability of replacement heifers and mean milk yield by the seasoned cow strongly dictates the age of culling. ^17^^,^^69^

### Milk production

In a commercial dairy farm, it is expected that a cow that is not yielding the anticipated quantities of milk is culled. Hence, it could be assumed that low producing cows are at high risk of culling as milk is the primary product. However, previous studies have reported that high producing cows are most likely to be culled than low producing cows due to the complications associated with high levels of production. ^18^^,^^42^ Complications such as recurrent milk fever and abortion are common reasons high milk producers are often culled. ^12^^,^^18^^,^^67^ Poor milk yield has been attributed to age, clinical mastitis and recurrent mastitis, and breed types. On the other hand, a rise in the culling rate of cows has often associated with an increase in milk yield. ^42^ There is no clear scientific explanation of this correlation between culling and high producing cows. However, several reports have highlighted that high milk yield is a contributing factor to the culling of dairy cows. ^12^^,^^13^^,^^24^^,^^25^ In addition to the ambiguity of why high milk producers are culled, there is a negative correlation between mastitis (a common reason for culling dairy cows) and high milk production. ^12^ The closest reasoning or influence to the culling of high producing cows is abortion cases, especially in Holstein breeds. Another reason for culling high producing cows is a significant drop in milk production. The decline in milk yield is influenced by age, nutrition, and diseases. ^12^^,^^29^

## Mitigation strategies to limit culling and mortality of dairy cows

Culling of dairy cows is not always an indication of adversities on the farm. For instance, dairy cows can be voluntarily culled to maintain herd size and generate profit from the sale of the surplus cows or heifers. Also, the culling of dairy cows is inevitable. It cannot be wholly eradicated; however, some factors that influence involuntary culling, such as reproduction failures and diseases, can be prevented or mitigated.

Reproduction failures, to some extent, can be avoided through sound management practices and upgrades like improving heat detection and efficient artificial insemination. ^27^ Even so, heat detection by a farmer cannot be as instinctive and accurate ^70^ as it could be when performed by the bull; hence there should be regular training and updating for this activity where artificial insemination is practised. ^71^

Also, one of the major contributors to reproductive failure is pathogens, such as *Brucella abortus,* which results in infertility and abortion. Therefore, surveillance of this pathogen needs to be prioritised at all stages of gestation. Different laboratory tests should be conducted because a serological test may yield negative results, whereas a culture test and a more sophisticated molecular test may be positive. ^72^ Thus, it is not recommended to rely on one type of test.

In addition, the feasibility of age (15 months old) at first insemination needs to be investigated. This is because the farm's economic reasons often influence the decision to inseminate at an early age. ^73^ More minor details such as matching the bull’s body frame with the 15 month old heifer are overlooked, and cases of dystocia are encountered. ^74^

### Systematic recording system of health factors

A systematic recording system of health records may help minimise misdiagnosis and premature culling of dairy cows. An earlier study alluded that an improved recording system should provide an epidemiological baseline for each health factor that may tamper with dairy cow’s production and reproduction performance. ^16^ This is essential considering that the decision to cull dairy cows voluntarily is the farmer’s prerogative. ^12^^,^^75^ The reliance on farmers' context to voluntarily cull a dairy cow may be subjective; hence, a systematic recording system is necessary. Systematic recording promotes the evaluation of regular health disorders. Also, data derived from a systematic recording system should be the basis of breeding plans to improve the longevity of dairy cows. ^16^

### Improvement of genetic evaluation and selection strategies

The performance of dairy cows is mainly influenced by genetics and the environment, particularly nutrition and management. Genetic evaluation and prediction of cow survival should form part of management strategies to lower culling and mortality rates. ^76^ However, genetic evaluation and selection should not only revolve around the milk production potential of the cow. ^68^ Genetic evaluation throughout the heifer rearing stage should also be prioritised to help predict cow survival. Genetic evaluation at the heifer level may limit premature culling and eliminate important traits as some functional traits are cumulative even though they have low heritability. ^76^

Over the years, selection for mainly milk production has proven unsustainable, especially with high producing dairy cows being reported as highly susceptible to mastitis, lameness and reduced length of reproductive life. ^30^ Also, when semen is selected, physical characteristics, such as good leg and feet, and condition of the genitals, precedes other considerations such as cow survival. ^70^

Furthermore, the influence of heat stress on dairy cows varies with breeds and stages of production because there are genetic traits of heat tolerance that vary with breeds. ^63^ Previous attempts to cross-breed indigenous cattle breeds with the exotic breed to mitigate heat stress have, however, resulted in reduced milk yield. ^3^^,^^61^ However, the level of reduction of milk yield due to cross-breeding is not extensively documented. As such, it is unclear whether this drop is of any significance.

### Provision of shelter, shades, and improvised feeding to limit heat stress

High ambient temperatures directly adversely influence the productivity of the cows to the extent that they can even contribute to the mortality of the cows. ^19^^,^^77^ This is because animals respond to high temperatures by reducing feed intake, altering respiration rate, and increasing water intake to facilitate cooling. This might have a limited impact over a single day or two during a hot summer’s day. However, under prolonged periods of high temperatures, the drop in feed intake becomes highly unsustainable, resulting in a decrease in milk yield volumes. ^3^^,^^61^ Hence, establishing shelters and shades around the farm would provide great relief to dairy cows and consequently reduce culling and mortality rates.

## Conclusion and recommendation

Reproduction failures have been singled out as the most common factor responsible for culling dairy cows in different countries. It is closely followed by production performance and mastitis as leading contributors to the decision to cull dairy cows. Dairy animals are culled because they are poor milk producers; even high milk producers are also culled due to abortion, lameness, and idiopathic reasons. As much as culling and mortality of dairy cows are inevitable, there is a need to revisit and revise dairy farming strategies, starting with the selection process and genetic evaluation. Functional traits should be considered if sustainability is to be achieved by the industry.

## Data availability

No data is associated with this article.
