# Supplementary material for: Culling and mortality of dairy cows: why it happens and how it can be mitigated
Source: F1000Res. 2022 Aug 1;10:1014. Originally published 2021 Oct 6. [Version 2] doi: 10.12688/f1000research.55519.2 (PMC9356254; doi:10.12688/f1000research.55519.2)
Supplement: Peer review history

# Peer review: Reviewer response for version 2

**DOI:** 10.5256/f1000research.133896.r146163

**Published:** 2022-08-05

**Authors:** Thankgod Onyiche

**Affiliations:** 1 Department of Veterinary Parasitology and Entomology, University of Maiduguri, Maiduguri, Borno, Nigeria

The authors have revised the manuscript in line with my earlier recommendations. Hence, the overall quality of the manuscript have greatly improved.

Is the review written in accessible language?

Yes

Are all factual statements correct and adequately supported by citations?

Yes

Are the conclusions drawn appropriate in the context of the current research literature?

Yes

Is the topic of the review discussed comprehensively in the context of the current literature?

Partly

Reviewer Expertise:

Veterinary science with more emphasis on parasitology

I confirm that I have read this submission and believe that I have an appropriate level of expertise to confirm that it is of an acceptable scientific standard.

# Peer review: Reviewer response for version 2

**DOI:** 10.5256/f1000research.133896.r146162

**Published:** 2022-08-02

**Authors:** Emmanuel Okechukwu Njoga

**Affiliations:** 1 Department of Veterinary Public Health and Preventive Medicine, Faculty of Veterinary Medicine, University of Nigeria, Nsukka, Nigeria

Thank you once more for inviting me for this review. The authors have addressed major issues raised. In my opinion, I think the paper is good to go. Therefore, I recommend indexing.

Is the review written in accessible language?

Yes

Are all factual statements correct and adequately supported by citations?

Yes

Are the conclusions drawn appropriate in the context of the current research literature?

Yes

Is the topic of the review discussed comprehensively in the context of the current literature?

Partly

Reviewer Expertise:

Veterinary Science and Public Health

I confirm that I have read this submission and believe that I have an appropriate level of expertise to confirm that it is of an acceptable scientific standard.

# Peer review: Reviewer response for version 1

**DOI:** 10.5256/f1000research.59100.r116136

**Published:** 2022-01-12

**Authors:** Thankgod Onyiche

**Affiliations:** 1 Department of Veterinary Parasitology and Entomology, University of Maiduguri, Maiduguri, Borno, Nigeria

**Introduction**

 This section can be further improved by highlighting and citing adequate literature on the current needs and demands for milk, not only using South Africa as reference, but other countries from different regions within Africa and the rest of the world.

 In line 2, page 3, could you kindly presents the statistics for the growth rate for 2017 at 3.9% before that of 2018 at 4.8%

**Literature search**

 It will be interesting for the authors to clearly state the search outcome from each of the databases used. For example, web of science (n=36). To allow for reproducibility, it will be good if the authors presents this search strategy using the standard protocol for the key terms used in the searches to be in inverted commas: for example, “Culling”. If Boolean operators were used when searching in the databases, then the authors could kindly state this: for example, “AND”, “OR”.

 Could they also provide additional information on the date where they last search was carried out. Articles are being published daily, and potential readers should be made sure that the search was conducted properly and adequately, so that no article was erroneously left out. 

**Diseases**

 With respect to diseases, to ensure orderly presentation of thoughts, the authors should discuss the various diseases broadly under two headings; infectious and non-infectious causes. Under the infectious causes, neosporiasis, mastitis *etc. *can be discussed.

 In your discussion for bovine neosporiasis, ketosis and milk fever, I would like to see a more in depth discussion, the present treatment is too short.

 In the last line of page 5, the expression “A similar trend was reported in similar studies” should be rephrased.

 In the general discussion of economic losses due to mastitis, the authors must be consistent in terms of the currency value: for example, some of the losses were expressed in Euros while others was in US dollars. This should be standardised. Nonetheless, I appreciate that the base was in South Africa rand.

 On page 7, under the sub theme “infertility”, the expression “Factors causing infertility and animals showing a diminished...' requires elaboration. What factors cause infertility? Please clarify.

 Due to paucity of studies in Africa on factors responsible for culling and mortality in farm animals, I will like to see how the authors relate their findings from studies in the western world to most probably what we have in Africa and the likely reasons why studies on this subject matter has not been published or brought to the front burner in this field.

Is the review written in accessible language?

Yes

Are all factual statements correct and adequately supported by citations?

Yes

Are the conclusions drawn appropriate in the context of the current research literature?

Yes

Is the topic of the review discussed comprehensively in the context of the current literature?

Partly

Reviewer Expertise:

Veterinary science with more emphasis on parasitilogy

I confirm that I have read this submission and believe that I have an appropriate level of expertise to confirm that it is of an acceptable scientific standard, however I have significant reservations, as outlined above.

# Response

**Published:** 2022-03-29

**Authors:** Ishmael Jaja

**Affiliations:** University of Fort Hare, South Africa

We thank you for taking time to read our paper. The comments and input by reviewers have greatly improved our paper, for this we are grateful.

 To Reviewer 2

**S/N**

**Reviewer’s comment**

**Authors’ response**

**Line number**

 1. This section can be further improved by highlighting and citing adequate literature on the current needs and demands for milk, not only using South Africa as reference, but other countries from different regions within Africa and the rest of the world.

 The section has been further improved with statistics and trends from other African countries.

 (p39-45)

 2. In line 2, page 3, could you kindly presents the statistics for the growth rate for 2017 at 3.9% before that of 2018 at 4.8%.

 Statistics for the growth from previous years have been included in the paragraph.

 (p27, 28)

 3.

 It will be interesting for the authors to clearly state the search outcome from each of the databases used. For example, web of science (n=36). To allow for reproducibility, it will be good if the authors presents this search strategy using the standard protocol for the key terms used in the searches to be in inverted commas: for example, “Culling”. If Boolean operators were used when searching in the databases, then the authors could kindly state this: for example, “AND”, “OR”.

 The authors appreciate the advice and promise to opt for systematic review in the future in which the suggested approach will be in effect. We, however, wish to keep this work as a traditional literature synthesis.

 N/A

 4

 Could they also provide additional information on the date where they last search was carried out. Articles are being published daily, and potential readers should be made sure that the search was conducted properly and adequately, so that no article was erroneously left out.

 The authors appreciate the advice and promise to opt for systematic review in the future in which the suggested approach will be in effect. We, however, wish to keep this work as a traditional literature synthesis.

 N/A

 5.

 With respect to diseases, to ensure orderly presentation of thoughts, the authors should discuss the various diseases broadly under two headings; infectious and non-infectious causes. Under the infectious causes, neosporiasis, mastitis etc. can be discussed.

 In your discussion for bovine neosporiasis, ketosis and milk fever, I would like to see a more in depth discussion, the present treatment is too short.

 The diseases have been divided into infectious and non-infectious diseases. Pathophysiology of the suggested diseases has been added to provide with more in-depth information.

 (p103, 125)

 6.

 In the last line of page 5, the expression “A similar trend was reported in similar studies” should be rephrased.

 The expression has been revised.

 (p216)

 7.

 In the general discussion of economic losses due to mastitis, the authors must be consistent in terms of the currency value: for example, some of the losses were expressed in Euros while others was in US dollars. This should be standardised. Nonetheless, I appreciate that the base was in South Africa rand.

 The currency for the losses due to culling has been corrected throughout the document.

 (p208 - 217)

 8.

 On page 7, under the sub theme “infertility”, the expression “Factors causing infertility and animals showing a diminished...' requires elaboration. What factors cause infertility? Please clarify.

 Factors contributing infertility are covered.

 311-315

 9.

 Due to paucity of studies in Africa on factors responsible for culling and mortality in farm animals, I will like to see how the authors relate their findings from studies in the western world to most probably what we have in Africa and the likely reasons why studies on this subject matter has not been published or brought to the front burner in this field.

 An account has been given at the end of the conclusion and recommendation.

 (p427-430)

# Peer review: Reviewer response for version 1

**DOI:** 10.5256/f1000research.59100.r96317

**Published:** 2021-10-28

**Authors:** Emmanuel Okechukwu Njoga

**Affiliations:** 1 Department of Veterinary Public Health and Preventive Medicine, Faculty of Veterinary Medicine, University of Nigeria, Nsukka, Nigeria

This paper is well written and scientifically sound. The topic addressed important livestock diseases that are prevalent in South Africa, and also provided the disease control measures. Once the issues raised below are addressed, this paper will be approvable without reservations.

**Introduction**

 First paragraph, 6th sentence: rewrite to read "The increased availability and access will enhance food security...".

 Second paragraph, 8th sentence: replace "point “with "points".

**Literature Search**

 Table 1:

 A review paper on the causes of mortality and culling in dairy cows in South Africa should identify, mention, discuss/review the causes in Africa first before comparing or mentioning the causes in other continents. The local problems/causes in Africa may be different from that of other continents and hence need to be discussed. If the causes are the same globally, then it should be listed in the table so that it will be evident to the scientific community.

 Therefore, the authors should include published papers from Africa in the Table. Some suggested ones are included here, for South Africa, ^1^ ^2^ Ethiopia, ^3^ ^4^ Nigeria, ^5^ Kenya, ^6^ and Senegal. ^7^

**Major Factors for Culling**

 Diseases: the authors could include brucellosis as this is a major problem in the dairy industry. The zoonotic and economic (production loss) importance of it need to be discussed.

 Milk fever and ketosis: can the authors provide few lines on the pathogenesis/pathophysiology of ketosis? This might aid readers in their understanding of the condition, especially among those outside the field of veterinary and animal sciences.

 Mastitis: third paragraph, what do the authors mean by “mechanical dynamics"?

 Can any of the pathogens listed be zoonotically transmitted via the handling of an infected cow or the drinking of contaminated and unpasteurized milk?

 Lameness: fourth sentence, replace "sensitive” with "importance". Second paragraph: lameness is not a disease but a condition.

 Heat stress: third sentence, delete "pregnancy “so that the sentence reads "... can not conceive".

Is the review written in accessible language?

Yes

Are all factual statements correct and adequately supported by citations?

Yes

Are the conclusions drawn appropriate in the context of the current research literature?

Yes

Is the topic of the review discussed comprehensively in the context of the current literature?

Partly

Reviewer Expertise:

Veterinary Science and Public Health

I confirm that I have read this submission and believe that I have an appropriate level of expertise to confirm that it is of an acceptable scientific standard, however I have significant reservations, as outlined above.

## References

[1] : A retrospective survey of the factors responsible for culling and mortality in dairy farms in the Eastern Cape Province, South Africa. *Scientific African* .2021;12: 10.1016/j.sciaf.2021.e00838 10.1016/j.sciaf.2021.e00838

[2] : Dairy farm-workers' knowledge of factors responsible for culling and mortality in the Eastern Cape Province, South Africa. *Trop Anim Health Prod* .2021;53(3) : 10.1007/s11250-021-02845-6 398 10.1007/s11250-021-02845-6 34250579PMC8273056

[3] : Assessment of the Reasons for Culling and its Relation to Age at Culling in Dairy Cows in and around Mekelle City, Tigray, Ethiopia. *Veterinary Medicine – Open Journal* .2021;6(1) : 10.17140/VMOJ-6-152 1-5 10.17140/VMOJ-6-152

[4] : A survey of management practices and major diseases of dairy cattle in smallholdings in selected towns of Jimma zone, south-western Ethiopia. *Animal Production Science* .2020;60(15) : 10.1071/AN19079 10.1071/AN19079

[5] : Recurrent Outbreaks of Lumpy Skin Disease and its Economic Impact on a Dairy Farm in Jos, Plateau State, Nigeria. *Nigerian Veterinary Journal* .2017;38(2) : Reference source

[6] : Impact of foot-and-mouth disease on mastitis and culling on a large-scale dairy farm in Kenya. *Vet Res* .2015;46: 10.1186/s13567-015-0173-4 41 10.1186/s13567-015-0173-4 25889460PMC4397692

[7] : Seroprevalence of Neospora caninum antibodies and its consequences for reproductive parameters in dairy cows from Dakar-Senegal, West Africa. *Trop Anim Health Prod* .2010;42(5) : 10.1007/s11250-009-9513-6 953-9 10.1007/s11250-009-9513-6 19997972

# Response

**Published:** 2022-03-29

**Authors:** Ishmael Jaja

**Affiliations:** University of Fort Hare, South Africa

We thank you for taking time to read our paper. The comments and input by reviewers have greatly improved our paper, for this we are grateful.

 Reviewer 1

**S/N**

**Reviewer’s comment**

**Authors’ response**

**Line number**

 1.

 First paragraph, 6th sentence: rewrite to read "The increased availability and access will enhance food security...".

 The 6 ^th^ sentence has been corrected and written as per advice

 (p31)

 2.

 Second paragraph, 8th sentence: replace "point “with "points".

 Point has been edited to points

 (p58)

 3.

 Table 1:

 A review paper on the causes of mortality and culling in dairy cows in South Africa should identify, mention, discuss/review the causes in Africa first before comparing or mentioning the causes in other continents. The local problems/causes in Africa may be different from that of other continents and hence need to be discussed. If the causes are the same globally, then it should be listed in the table so that it will be evident to the scientific community.

 Therefore, the authors should include published papers from Africa in the Table. Some suggested ones are included here, for South Africa, ^1^ ^2^ Ethiopia, ^3^ ^4^ Nigeria, ^5^ Kenya, ^6^ and Senegal. ^7^

 An African perspective of culling and mortality has been duly incorporated in the work and table has been updated.

 (p86, 119)

 4.

 Diseases: the authors could include brucellosis as this is a major problem in the dairy industry. The zoonotic and economic (production loss) importance of it need to be discussed.

 Brucellosis has been added and discussed in the review paper as per the reviewer’s advice.

 (p111-124)

 5.

 Milk fever and ketosis: can the authors provide few lines on the pathogenesis/pathophysiology of ketosis? This might aid readers in their understanding of the condition, especially among those outside the field of veterinary and animal sciences.

 Brief pathogenesis of both milk fever and ketosis has been added to the review as per advice.

 (p127-172)

 6.

 Mastitis: third paragraph, what do the authors mean by “mechanical dynamics"?

 The paragraph has been rephrased thus removing mechanical dynamics.

 (p183)

 7.

 Lameness: fourth sentence, replace "sensitive” with "importance".

 Sensitive has been replaced with importance/ important.

 (p226, 227)

 8.

 Second paragraph: lameness is not a disease but a condition.

 In instances whereby, lameness has been referred to as a disease instead of condition, corrections have been made.

 (p223)

 9.

 Heat stress: third sentence, delete "pregnancy “so that the sentence reads "... can not conceive".

 Pregnancy has been removed

 (p287)
